# Pestilence and Plague

**Published:** 1877-04

**Authors:** 


### *CANCER.*

The readers of the JOURNAL will remember that for some years past we have maintained that cancer, before arriving at the period of ulceration, is generally curable. Our belief is founded on cases that have come under our observation, where the eradication of the tumor has been effected by outward applications. But we were hardly prepared to believe that the disease could be expelled from the system, by any plan of treatment, after the period of ulceration and blood poisoning. And if the statement had come to us from an irresponsible source, we should not have heeded it. But when proclaimed by so competent an authority as Dr. Rae—the pioneer in "Medical Electricity," and probably the most competent electrician in this country—his statement is worthy of serious consideration, and we rejoice in the promise it offers to those who are afflicted with this unfortunate disease.

### *CANCER CURED BY ELECTRICITY AND HOMŒOPATHY.*

BY DR. J. H. RAE, Office, 51 Bible House, New York.

At the time the cure was effected, and several times since, I have been urged by friends and homœopathic physicians to publish a history of the case for the benefit of the profession, but business matters and a long absence in the Rocky Mountains prevented my doing so.

This case is peculiar, and at the time of cure, 1869, created considerable excitement. What gave prominence to it was that H. Marion Sims, the eminent surgeon, twice operated surgically, each time removing the tumor with the knife. The first operation, removing the tumor from left breast, was performed in Paris in 1868, after English and French surgeons had pronounced it too dangerous for them to attempt; the second operation, removing tumor from under left arm, was accomplished in February, 1869, at the St. Cloud Hotel, New York City, by Dr. H. M. Sims, assisted by Dr. Nott; thus the character of the tumors is well authenticated. Upon the appearance of the third tumor on the right breast, Mr. Davis being by this time too reduced physically to admit of a third surgical operation, was advised to try electricity. Dr. Neftel, a Russian physician and electrician, was applied to, and as Mr. Davis afterward informed me, performed several operations without much, if any, effect; after this, I believe, Dr. Neftel applied electrolysis or puncture, but this was very weakening, or supposed to be so, and Mr. Davis came to Syracuse. Upon his arrival he and his friends urged me to undertake the case. I hesitated some time, thinking that I might possibly be interfering with Dr. Neftel, but upon Mr. Davis' assurance that it would not, I consented, and give below the facts of treatment :

May 12th, 1869. Made examination; found tumor about the size of a large hen's egg, shaped like a bean, just below and to the right of the nipple on the right breast; the tumor exhibited several small red spots, where the electrolysis had been applied; the case being so well known I made no further diagnosis, but proceeded at once to operate.

I used the *primary* current, half strength, passing it through *silver electrodes made for this case.* The negative electrode was placed on the breast, about two inches from the tumor; the positive I placed upon various parts of the tumor, moving the negative to correspond with the moving of the positive; operated about twelve minutes; the patient exhibiting signs of exhaustion, I stopped.

Before applying the electricity, I made the skin moist with tincture Thuja occidentalis, reduced one-half with water. I gave one pellet No. 35, Aconite$^{3d}$. *Same evening* gave another operation, this time using the superinduced or attenuated current, giving what I denominate constitutional treatment, applying the negative at the lower extremity of the spine (coccyx), manipulating with positive the entire length of the spine, also over the breast and stomach, finishing with a few passes on the head. Gave one pellet Nux, same as Aconite.

May 13th. Repeated operations same as day before; gave no medicine.

May 14. Repeated operation, giving medicine same as first day.

May 15th. On this day, after operating, made a close examination of tumor; the nurse and myself both satisfied that the tumor was softer, less hardness of skin, and we thought it had decreased some, but the patient was stronger, had better appetite, and was in good spirits. Operated twice as usual.

Kept up treatment as described, giving the two remedies every other day until about 1st of June; patient growing stronger daily.

June 1st. Upon a close examination of tumor, the patient, nurse, and myself became satisfied that the tumor had diminished in size ONE-FOURTH, at least; the patient was affected to tears (but tears of joy); the nurse and two or three friends perfectly astonished. We all now felt that I had control of the disease, and were satisfied that I should effect an ultimate cure. Precisely the same treatment of electricity and medicine kept up until about the 1st of August. Patient became comparatively strong, increased several pounds in flesh. At this time patient went to New York upon business, and while there was examined by his former physicians, and Mr. Davis reported to me that they pronounced the cancer radically cured.

Above you have a true history of this case. I make no comments, offer no theory, but give a plain statement of facts, and leave the readers to form their own conclusions.

The Magnetic Machine used was Dr. Rae's Three Current Machine, now for sale for the first time.

